# Land use and soil characteristics affect soil organisms differently from above-ground assemblages

**DOI:** 10.1186/s12862-022-02089-4

**Published:** 2022-11-17

**Authors:** Victoria J. Burton, Sara Contu, Adriana De Palma, Samantha L. L. Hill, Harald Albrecht, James S. Bone, Daniel Carpenter, Ronald Corstanje, Pallieter De Smedt, Mark Farrell, Helen V. Ford, Lawrence N. Hudson, Kelly Inward, David T. Jones, Agnieszka Kosewska, Nancy F. Lo-Man-Hung, Tibor Magura, Christian Mulder, Maka Murvanidze, Tim Newbold, Jo Smith, Andrew V. Suarez, Sasha Suryometaram, Béla Tóthmérész, Marcio Uehara-Prado, Adam J. Vanbergen, Kris Verheyen, Karen Wuyts, Jörn P. W. Scharlemann, Paul Eggleton, Andy Purvis

**Affiliations:** 1 Science and Solutions for a Changing Planet DTP, and the Department of Life Sciences, Imperial College, London, UK; 2 Natural History Museum, London, UK; 3 United Nations Environment Programme World Conservation Monitoring Centre, Cambridge, UK; 4 Technische Universitaet Muenchen, Chair of Restoration Ecology, Freising, Germany; 5 Environmental Resources Management (ERM) Limited, London, UK; 6 Cranfield University, Cranfield, UK; 7 Forest and Nature Lab, Department of Environment, Ghent University, Gontrode (Melle), Ghent, Belgium; 8 CSIRO Agriculture and Food, Glen Osmond, Kaurna Country Australia; 9 Bangor University, Bangor, UK; 10 Department of Entomology, Phytopathology and Molecular Diagnostics, University of Warmia and Mazury in Olsztyn, Olsztyn, Poland; 11 Laboratory of Gene Expression and Evolution in Arthropods, Department of Genetics and Evolutionary Biology, University of São Paulo, São Paulo, Brazil; 12 Department of Ecology, Faculty of Science and Technology, University of Debrecen, Debrecen, Hungary; 13 ELKH-DE Anthropocene Ecology Research Group, University of Debrecen, Debrecen, Hungary; 14 Department of Biological, Geological and Environmental Sciences, University of Catania, Catania, Italy; 15 Faculty of Exact and Natural Sciences, FI. Javakhishvili Tbilisi State University, Tbilisi, Georgia; 16 Centre for Biodiversity and Environment Research, Department of Genetics, Evolution and Environment, University College London, London, UK; 17 MV Agroecological Research Centre PT, Mértola, Portugal; 18 Department of Evolution, Ecology and Behavior and Department of Entomology, University of Illinois, Urbana, USA; 19 Wildlife Conservation Society, Indonesia Program, Bogor, Indonesia; 20 MTA-DE Biodiversity and Ecosystem Services Research Group, Debrecen, Hungary; 21 Independent Researcher, Campinas, São Paulo Brazil; 22 Agroécologie, INRAE, Institut Agro, Univ. Bourgogne, Univ. Bourgogne Franche-Comté, 21000 Dijon, France; 23 Lab of Environmental and Urban Ecology, Research Group Environmental Ecology and Microbiology (ENdEMIC), Department of Bioscience Engineering, University of Antwerp, Antwerp, Belgium; 24 School of Life Sciences, University of Sussex, Brighton, UK; 25 Department of Life Sciences, Imperial College London, Silwood Park, Ascot, UK

**Keywords:** Land-use, Land-use intensity, Soil biota, Soil biodiversity, Organism abundance, Mixed-effects models

## Abstract

**Background:**

Land-use is a major driver of changes in biodiversity worldwide, but studies have overwhelmingly focused on above-ground taxa: the effects on soil biodiversity are less well known, despite the importance of soil organisms in ecosystem functioning. We modelled data from a global biodiversity database to compare how the abundance of soil-dwelling and above-ground organisms responded to land use and soil properties.

**Results:**

We found that land use affects overall abundance differently in soil and above-ground assemblages. The abundance of soil organisms was markedly lower in cropland and plantation habitats than in primary vegetation and pasture. Soil properties influenced the abundance of soil biota in ways that differed among land uses, suggesting they shape both abundance and its response to land use.

**Conclusions:**

Our results caution against assuming models or indicators derived from above-ground data can apply to soil assemblages and highlight the potential value of incorporating soil properties into biodiversity models.

**Supplementary Information:**

The online version contains supplementary material available at 10.1186/s12862-022-02089-4.

## Background

Terrestrial biodiversity continues to decline globally in the face of increasing human impacts [1], with land-use change and intensification the biggest driver of recent biodiversity loss [2]. Species extinction rates are estimated to be around 10–1000 times higher than the background rate [3], with 1 million plant and animal species threatened with extinction and the Living Planet Index (which reflects trends in vertebrate population size) declined by 69% between 1970 and 2022 [4]. However, these assessments and indicators focus on data-rich taxa, especially vertebrates, and so may not reflect broader biodiversity patterns [5].

Organisms that live in the soil and leaf litter (henceforth, soil biodiversity) are particularly poorly represented in indicators and assessments of the global state of nature [6, 7]. This is despite the fact that they comprise 23% of described living species, support ecosystem services, such as nutrient cycling, soil formation and water quality [8, 9], valued at $2.1 trillion per year worldwide [10] and form the second largest carbon pool on Earth [11]. This poor representation partly reflects data limitations: taxonomic discovery is less complete for many groups of soil species than those above ground, their distributions are less well known, and their assemblage structure is less often quantified [12, 13]. Additionally, because soil biodiversity samples are often not identified to the species level and because soil-dwelling species may be more taxonomically inclusive (‘lumped’) than above-ground species [14, 15], estimates of diversity may not be comparable with those for better-known taxa. Although soil and above-ground communities are linked mechanistically [8, 16], they often show different patterns of diversity [17, 18]. Soil characteristics can also affect biodiversity within both soil and above-ground assemblages. Soils are a fundamental determinant of plant communities [19], with soil biota being linked to them directly through symbiosis and herbivory, and indirectly via decomposition and nutrient cycling [20], but global patterns of soil fauna biomass may not follow plant biomass [21].

**Fig. 1:**
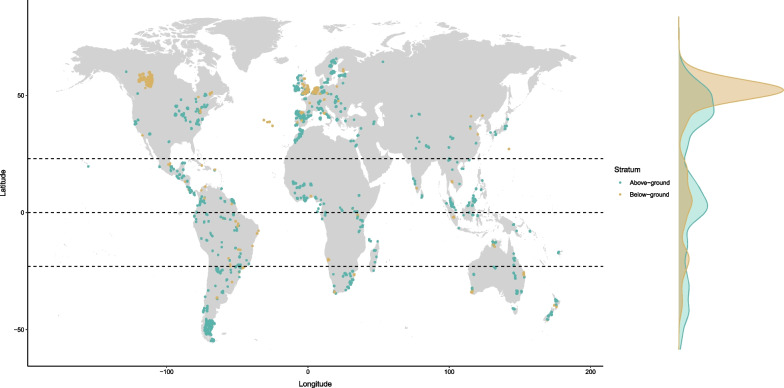
Locations where above-ground (green points, 19,651 sites/locations) and soil (orange points, 7155 sites/locations) biodiversity were sampled. The density plot shows the latitudinal distribution of above-ground (green) and soil (orange) sites

We analyse biodiversity data [22, 23] from 19,651 above-ground and 7155 soil assemblages (comprising vertebrates, invertebrates, plants, and fungi) (Table 1) in different land uses worldwide (Fig. 1 and Table 2), alongside global datasets of soil characteristics [24]. Because soil assemblage data are often less taxonomically precise than data from above-ground assemblages, the response variable we model is the summed abundance of all taxa sampled. This measure is very much less sensitive to change than more information-rich measures that incorporate species identity [25], but it has the advantage that significant differences between models cannot be artefacts of differences in taxonomic precision. The Soil Biodiversity Observation Network (SoilBON) have proposed population abundance as an Essential Biodiversity Variable [7].

**Table 1:** Summary of soil biodiversity and above-ground biodiversity data included in this analysis

	Above-ground	Soil
Sources	412	101
Studies	498	122
Sites	20,178	7515
Countries	88	37
Biomes	14	13
Total taxa	55,443	13,056
Vascular plants	19,166	0
Bryophytes	1454	0
Fungi and Mycetozoa	1464	479
Vertebrates	9704	3
Annelids	5	401
Arthropods	23,194	11,606
Other invertebrates	5	6
Molluscs	451	34
Nematodes	0	527

Sources typically represent a single paper with each source containing one or more Studies, defined as data collected using the same sampling method. Sites are individual sampling points

**Table 2:** Sites by land use and use intensity for soil biodiversity and above-ground biodiversity based on the description of the Source authors

Land Use	Above-ground biodiversity			Soil biodiversity		
	Minimal	Light	Intense	Minimal	Light	Intense
Primary vegetation	4326	2153	491	826	395	121
Secondary vegetation	2512	1364	502	655	341	236
Plantation forest	552	1238	357	153	198	152
Cropland	567	1081	1121	143	288	331
Pasture	873	1185	327	726	1085	1146

For more details on curation see Additional file 1: Tables S3, S4 and Hudson et al. [22]

**Fig. 2:**
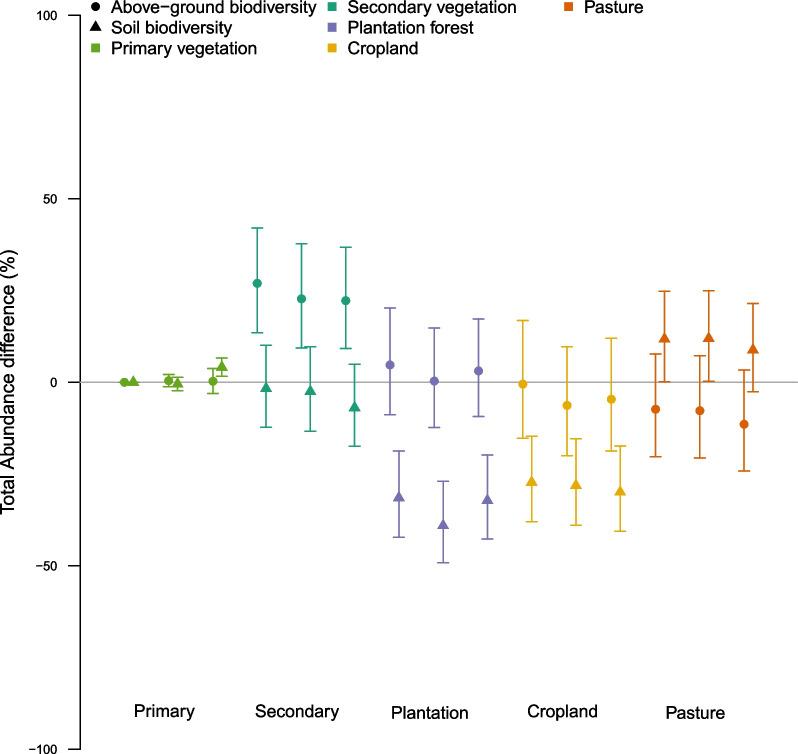
Response of above-ground (circles) and soil (triangles) organismal abundance to land-use type and intensity (from left to right within each land use: minimal, light, and intense use) compared to abundance in primary vegetation (baseline). Responses have been back-transformed. For this plot, other fixed effects are set at their median values. Error bars show 95% confidence intervals

To accommodate heterogeneity due to the wide range of sampling methods and macroecological gradients in the dataset we used mixed-effects models to test three main hypotheses (expanded on in Additional file 1: Table S1): (1) Because some land uses imply very different levels of perturbation to soil versus above-ground microenvironments, and because soil organisms are less mobile and more sensitive to microclimate change [26], we expect differences in how assemblages from these two settings respond to land use. For example, using land to rear livestock may impact soil structure much less than above-ground habitat structure, while soil organisms may take longer than above-ground taxa to recolonise sites recovering from physical soil disturbance [27–29]. (2) Because physical properties of soil, such as pH and soil texture, themselves mediate the impacts of land use microenvironments, we expect these properties to influence assemblage responses to land use. For instance, moisture retention by clay-rich soils may mitigate the warming and drying effects of agriculture. (3) Although above-ground and soil assemblages are linked mechanistically, we do not expect soil properties to shape their assemblage-level responses to land use in the same way.

To offset the different geographic biases in soil versus above-ground assemblages we ran a weighted model. Weights were calculated by dividing the number of soil sites by the number of above-ground sites within each biome. In addition to the models required to test the three hypotheses, we also constructed a set of simpler models with single or additive terms to fully characterise which terms in the full model contributed the most explanatory power. We also undertook two sensitivity analyses. Because the biome-weighting is not commonplace (despite the ubiquity of geographic biases in biodiversity databases [30]), our first sensitivity analysis repeated the modelling without it. The second sensitivity analysis addresses the point that soil and above-ground assemblage data sets obviously have very different compositions in terms of which major taxonomic groups are well represented. While different impacts of land use on soil and above-ground assemblages would still be important even if they simply reflected such taxonomic differences, we also ran the same models using only the data for invertebrate taxa.

## Results

Estimated effects of land use differed markedly between soil and above-ground biota (Table 3); relative to primary vegetation, soil assemblages had lower abundance than above-ground assemblages in secondary vegetation and (especially) plantation forest and cropland, but higher abundance in pasture (Fig. 2).

**Fig. 3:**
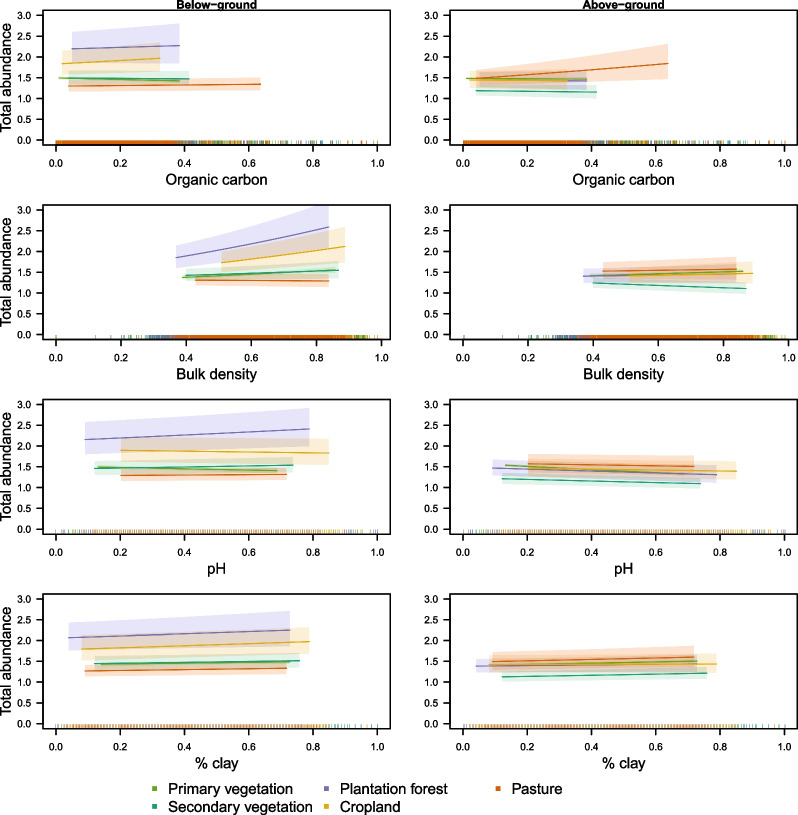
The (back-transformed) response of abundance to soil properties for five land uses for soil biodiversity (left) and above-ground biodiversity (right), with median values for other fixed effects. Shading spans ± 0.5 standard errors, and rugs along the x axes show the values of the explanatory variables represented in the data set used for modelling

**Table 3:** Model comparison table for the three models used for hypothesis testing compared with the full model, for all taxa and invertebrate only subset

		All taxa			Invertebrates only		
Model	d.f.	AIC	log lik.	$\chi ^2$	AIC	log lik.	$\chi ^2$
Full model	73	− 12949.60	6547.80		− 9616.36	4881.18	
Full model minus UI	53	− 13244	6675.3	145.35	− 9615.97	4860.99	171.06
Full model minus LUI × habitat layer interaction (hypothesis 1)	30	− 13284.10	6685.05	125.78	− 9896.90	4991.45	139.49
Full model minus soil property × land use interactions (hypothesis 2)	32	− 13121.93	6601.96	291.96	− 9841.82	4961.91	198.58
Full model minus soil property × land use × habitat layer interactions (hypothesis 3)	20	− 13262.79	6684.40	127.10	− 9931.08	5018.54	85.31

All results were significant at p < 0.001

Soil properties, especially bulk density, affected how soil fauna abundance responds to land use (Fig. 3, Table 3). These effects were not consistent among land uses; for example, abundance correlated positively with bulk density among cropland and plantation sites but not among pasture sites (Fig. 3). Soil properties also mediated the responses of above-ground assemblages to land use, in ways that differed from how they shaped the responses of soil assemblages (Table 3). Like the soil biota, effects on above-ground biodiversity were not consistent among land uses, e.g., the positive correlation between above-ground organism abundance and organic carbon was more pronounced in cropland and pasture than in other land uses (Fig. 3).

As expected with such heterogeneous data, most of the explained variation was attributed to random effects; but interactions increased the explanatory power of the fixed effects by nearly half, from 14% to 20% (Additional file 1: Fig. S1). The unweighted model found broadly similar patterns between above-ground and soil assemblage responses to land use but had lower explanatory power (see Additional file 1). Compared to the pattern shown in Fig. 2, analysis of the invertebrate-only data found a bigger difference in the effects of plantations on soil versus above-ground assemblages, but a negligible difference in how cropland affected them (see Additional file 1).

## Discussion

Land use—the recent main driver of biodiversity loss worldwide [1, 2]—affects soil assemblages differently from those above ground. As hypothesised, cropland—where tillage, pesticides and fertilisers disturb soil biodiversity [31, 32]—reduces abundance even more in the soil than above ground. In contrast, pasture—with relatively little physical disturbance of the soil and often increased nutrient input [33, 34]—shows the opposite pattern. Clear-felling and replanting with different tree species has been previously found to have the strongest negative impact on biodiversity [35], and we also find a strong negative effect of plantation on biodiversity. The much lower relative abundance in soil than above-ground assemblages (Fig. 2) may be explained by the acidified soil and recalcitrant leaf litter typical of conifer plantations [36] (the dominant type in the soil assemblage data), together with drier soils in plantations that have a reduced under-story [26]. Soil organism abundance has not recovered in secondary vegetation as much as above-ground abundance (Fig. 2), in keeping with our hypothesis that soil biota recovers more slowly to disturbance than above-ground biodiversity.

As well as affecting the overall abundance of soil organisms, soil properties also mediated how land use affected soil assemblages (left-hand column of Fig. 3). Perhaps more surprisingly, soil properties also affected how above-ground assemblages responded to land use, in ways that differed from their effect on the responses of soil assemblages (right-hand column of Fig. 3, Table 3). Above-ground abundance generally increased with organic carbon, which is as expected given the latter’s close link with plant productivity [37]. Abundance was generally higher in more clay-rich soils, which typically have more nutrients and retain water better [38]. These effects of soil properties will include both direct, and indirect effects medicated by biotic interactions, but we were unable to separate these as few studies collected data on above-ground and soil biota concurrently.

The greater impact of cropland and plantation forestry on soil biota than above-ground assemblages shown by our models is a cause for serious concern. To feed the growing population, scenarios include the world’s croplands increasing in area, being managed more intensively, or both [39]. The rapid recent expansion of plantation forests may accelerate further if they receive subsidies for carbon sequestration, despite their impacts on biodiversity [40]. Pathways to sustainable development must avoid the diminution of soil assemblages that would undermine the long-term provision of soil ecosystem services [1]. This highlights the likely importance of soil biodiversity for the ecological intensification of agriculture [41] and of considering soil biodiversity explicitly in formulating conservation policy [7].

Our division of assemblages into soil and above-ground was based on how they were sampled rather than on ecosystem ecology. Many organisms sampled by above-ground methods spend part of their life cycle in the soil (e.g., many flies, bees and beetles), or even have much of their biomass underground (e.g., most plants). The soil assemblages have a very different taxonomic composition from those above-ground (Table 1) and different taxonomic groups are expected to respond differently to land use and soil properties [15, 42, 43]. An example of this can be seen in the invertebrate-only results (see Supplemental Information), here both above-ground and soil invertebrates are equally impacted in cropland, but above-ground invertebrate abundance is greater than soil invertebrates in plantation sites. Further work with models incorporating functional traits robust to coarse taxonomic resolution would be valuable. Better documentation of the taxonomic and functional diversity of soil fauna would also help overcome some of these limitations, so we echo calls for better soil biodiversity information systems [6, 44]. Additionally, except for fungi, micro-organisms are unrepresented in both soil and above-ground datasets—assemblage data from metabarcoding and metagenomic approaches [7, 45] will enable the use of more information-rich biodiversity measures.

Above-ground and soil taxa may be active and sampled at very different spatial scales, and soil property data with a spatial resolution of 250 m used here may not accurately reflect that experienced by the biota. Site-specific soil property data were available for some studies used in this analysis but were too insufficient or inconsistent to incorporate. Better standardisation of soil biodiversity surveys with a minimum level of environmental measurements collected would be a valuable contribution to the field [9, 46] as would explicit tests of spatial and temporal heterogeneity [47]. Likewise, models that consider other drivers, such as climate change, alongside land use will also improve understanding. Correlative models such as ours are sufficient for developing indicators and models for monitoring and combating biodiversity loss but there is also a need for an improved understanding of the mechanisms linking land use, soil properties, and biodiversity responses [8, 48]. Future analysis of this dataset using structural equation models (SEMs) could be used towards this, to disentangle the direct and indirect effects of soil properties and land use on communities. However, the limitations of our data and models do not detract from the central implication that soil and above-ground assemblages respond differently to land use: inferences drawn from what lives above ground cannot safely be extended to the soil biota.

## Conclusions

We show that soil biodiversity does not respond the same way to land use and soil properties as above-ground assemblages. The most widely used indicators of biodiversity, e.g. the Red List Index [49] and the Living Planet Index [50], include few or no soil taxa [6]. This means that current indicators, models, and frameworks for monitoring and combating biodiversity loss may be insufficient to safeguard the soil biodiversity needed to underpin ecosystem function.

## Methods

### Biodiversity data

In the absence of a well-developed catalogue of global soil biodiversity [44], we initially searched within the PREDICTS database [22, 23] for soil assemblage data, defining soil assemblages as those sampled within the soil; at the soil surface, or in the leaf litter. The database is a global compilation of studies that have each compared non-cultivated species assemblages at multiple sites facing different land-use and related pressures [22]. To the 59 studies (from 38 source publications with 1356 sites and 1570 taxa) of soil assemblage data previously in the PREDICTS database, we added 46 further studies (from 25 sources with 2726 sites and 3857 taxa (Tables 1 and 2 ). Above-ground assemblage data came from the other 509 studies (from 422 source publications, with 20,634 sites and 22,721 taxa) in the PREDICTS database at that time (October 2016).

The fraction of taxa resolved to species level was over twice as high in the above-ground assemblages as in the soil assemblages (58% versus 28%). Given this, plus the likelihood that species-level taxa are more inclusive in soil than above-ground organisms (i.e., the latter tend to be subdivided more finely when species are demarcated [15]), measures that use compositional information (such as diversity indices, or even numbers of species) cannot be compared between soil and above-ground assemblages. We therefore used the summed abundance across all sampled taxa—which is unaffected by taxonomic precision—as the site-level response variable. Whenever sampling effort varied among sites within a study, any abundance data sensitive to it (i.e., metrics not already reported as numbers per unit time, distance, area, or volume) were divided by sampling effort. Finally, abundance values were rescaled within each study to have a maximum value of 1, reducing among-study heterogeneity and thereby aid model convergence.

### Explanatory variables

Using the information in the original papers, each site was classified into one of six categories of land use—primary vegetation, secondary vegetation, plantation forest, cropland, pasture or urban—and either low, medium, or high use intensity (see Additional file 1 and [22] for full definitions). Most combinations of land use and use intensity (henceforth, LUI) had large enough sample sizes in both the above-ground and soil subsets, but even after targeted literature searching to augment the database’s holdings of urban data, there were insufficient sites for robust comparison of above- and below-ground in urban land use sites. The above-ground subset comprised primarily arthropods, plants, and vertebrates whereas the soil biodiversity subset was mostly arthropods (Table 1).

Nine soil properties widely reported to influence soil biodiversity (Additional file 1: Table S1) were obtained from the SoilGrids250m database [24] using ESRI ArcGIS 10.3 [51]. Values were not available for 49 sites, which were therefore removed from the analysis. We averaged the values from depths 0, 5, 15 and 30 cm as no biodiversity data sources sampled at depths greater than 30 cm. The soil properties were expected to be collinear so, before model construction began, generalised variance inflation factors (GVIFs) were calculated [52]. Among the soil texture properties, the percentage of clay had the lowest GVIF so was chosen in preference to percentages of silt or sand. Successively dropping the variable with the highest GVIF until all remaining GVIFs were low enough to suggest collinearity was not a major issue (all GVIF < 1.5), led to all soil moisture properties being dropped, while pH, bulk density, organic carbon, and clay percentage were retained.

### Biome weighting

To offset the geographic bias in soil versus above-ground assemblages (Fig. 1), we applied weights in the models. Weights were calculated by dividing the number of soil sites by the number of above-ground sites within each biome (Additional file 1: Table S2). Weights were calculated separately for the invertebrate-only subset (not shown).

### Statistical analysis

All analyses were carried out in R 3.5.1 [53]. Because total abundance contained non-integers even before rescaling, it was log(x + 1) transformed before modelling with Gaussian errors. The studies in the dataset vary widely in many aspects of sampling. We therefore fitted mixed-effects models (as implemented in *lme4* version 1.1.18.1 [54] with *bobyqa* numerical optimisation) to reduce heterogeneity caused by among-study differences in sampling methodology and macroecological gradients such as latitude.

Our previously listed hypotheses were tested by comparing a maximally complex model with three simpler models (see Additional file 1 for model structures) that lacked the hypothesised effects. The full model included six main fixed effects—land-use type and intensity (LUI), above-ground or soil assemblage (habitat layer), and the four soil properties (rescaled to the range 0-1 to aid fitting)—plus each soil property’s interaction with land-use type and habitat layer, the interaction between land-use type and habitat layer, and the three-way interactions of each soil property with land-use type and habitat layer. The random-effects structure was chosen by comparing the Akaike’s Information Criterion (AIC) of models having the full set of fixed effects plus, as random intercepts, (a) spatial block nested within study identity, (b) spatial block identity, or (c) study identity [52]; models with random slopes did not converge. The optimal random-effects structure was then retained for all models. To ascertain the influence of use intensity on the full model was compared to one with only land use. To test whether above-ground and soil assemblages respond differently to land use, the full model was compared to one in which habitat layer could not interact with other explanatory variables. The importance of soil properties in shaping assemblage responses to land use was tested by comparing the full model to one in which soil properties were included as main effects but could not interact with LUI. Whether effects of soil properties differ for above-ground and soil assemblages was tested by comparing the full model to one in which neither the soil properties nor their interactions with land use could interact with habitat layer.

In addition to the models required to test the three hypotheses, we also constructed a set of simpler models with single or additive terms to fully characterise which terms in the full model contributed most explanatory power. The variance explained by fixed effects alone (marginal R^2^_glmm_) and fixed and random effects combined (conditional R^2^_glmm_) were calculated using the *MuMIn* package [55] as measures of explanatory power. The random effects and spatial blocks intended to accommodate the heterogeneity among studies are expected to explain much more of the variance than the fixed effects. Consequently, when comparing models in terms of the explanatory power of their fixed effects, we compare their marginal R^2^_glmm_ / (1 - conditional R^2^_glmm_). We repeated the analyses without the weighting procedure used to compensate for the different geographic biases of above-ground and soil assemblage data (results in Additional file 1).

## Supplementary Information

**Additional file 1.** Contains Tables S1-S4 and Figures S1-S6, providing further information on data sources, model structures and results of sensitivity analyses.

## Data Availability

The datasets generated and/or analysed during the current study are available in The Natural History Museum Data Portal, https://doi.org/10.5519/zall06xc.
