# Supplementary material for: Land use and soil characteristics affect soil organisms differently from above-ground assemblages
Source: BMC Ecol Evol. 2022 Nov 17;22:135. doi: 10.1186/s12862-022-02089-4 (PMC9673366; doi:10.1186/s12862-022-02089-4)
Supplement: Supplementary file 1 — Additional file 1. Contains Tables S1-S4 and Figures S1-S6, providing further information on data sources, model structures and results of sensitivity analyses. [file 12862_2022_2089_MOESM1_ESM.pdf]

Land use and soil characteristics affect soil organisms differently from above-ground assemblages - supplemental information

Victoria J. Burton*[1,2], Sara Contu[2], Adriana De Palma[2], Samantha L. L. Hill[3], Harald Albrecht[4], James S. Bone[5], Daniel Carpenter[2], Ronald Corstanje[6], Pallieter De Smedt[7], Mark Farrell[8], Helen V. Ford[9], Lawrence N. Hudson[2], Kelly Inward[2], David T. Jones[2], Agnieszka Kosewska[10], Nancy F. Lo-Man-Hung[11], Tibor Magura[12,13], Christian Mulder[14], Maka Murvanidze[15], Tim Newbold[16], Jo Smith[17], Andrew V. Suarez[18], Sasha Suryometaram[19], Béla Tóthmérész[20], Marcio Uehara-Prado[21], Adam J. Vanbergen[22], Kris Verheyen[7], Karen Wuyts[23], Jörn P. W. Scharlemann[24], Paul Eggleton[2], and Andy Purvis[2,25]

[1]Science and Solutions for a Changing Planet DTP, and the Department of Life Sciences, Imperial College London, UK

[2]Department of Life Sciences, Natural History Museum, Cromwell Road, London SW7 5BD, UK

[3]United Nations Environment Programme World Conservation Monitoring Centre, 219 Huntingdon Road, Cambridge CB3 0DL, UK

[4]Technische Universitaet Muenchen, Chair of Restoration Ecology, Emil-Ramann-Str. 6, 85350 Freising, Germany

[5]Environmental Resources Management (ERM) Limited, London, UK

[6]Cranfield University, College Road, Cranfield, Bedfordshire, MK43 0AL, UK

[7]Forest and Nature Lab, Department of Environment, Ghent University, Geraardsbergsesteenweg 267, 9090 Gontrode (Melle), Belgium

[8]CSIRO Agriculture and Food, Locked Bag 2, Glen Osmond, SA 5064, Australia

[9]Bangor University, Bangor, Gwynedd, LL57 2DG, UK

[10]Department of Entomology, Phytopathology and Molecular Diagnostics, University of Warmia and Mazury in Olsztyn, Prawochenskiego 17, 10-719 Olsztyn, Poland

[11]Laboratory of Gene Expression and Evolution in Arthropods, Department of Genetics and Evolutionary Biology, University of São Paulo, Rua do Matão, 277, Cidade Universitária, Butantã, CEP. 05508-09, São Paulo, SP, Brazil

[12]Department of Ecology, Faculty of Science and Technology, University of Debrecen, Egyetem sq. 1, H-4032 Debrecen, Hungary

[13]ELKH-DE Anthropocene Ecology Research Group, University of Debrecen, Egyetem sq. 1, H-4032 Debrecen, Hungary

[14]Department of Biological, Geological and Environmental Sciences, University of Catania, Catania, Italy

[15]Faculty of Exact and Natural Sciences, I. Javakhishvili Tbilisi State University. Chavchavadze ave. 1. 0128 Tbilisi, Georgia
[16]Centre for Biodiversity and Environment Research, Department of Genetics, Evolution and Environment, University College London, Gower Street, London WC1E 6BT, UK
[17]MV Agroecological Research Centre, Moinhos de Vento, 7750-217 - Espirito Santo, Portugal
[18]Department of Evolution, Ecology and Behavior and Department of Entomology, University of Illinois, USA
[19]Wildlife Conservation Society - Indonesia Program, Bogor Tengah, Bogor 16151, Indonesia
[20]MTA-DE Biodiversity and Ecosystem Services Research Group, Egyetem tér 1, 4032 Debrecen, Hungary
[21]Independent researcher
[22]Agroécologie, INRAE, Institut Agro, Univ. Bourgogne, Univ. Bourgogne Franche-Comté, F-21000 Dijon, France
[23]Lab of Environmental and Urban Ecology, Research Group Environmental Ecology and Microbiology (ENdEMIC), Department of Bioscience Engineering, University of Antwerp, Groenenborgerlaan 171, 2020, Antwerp, Belgium
[24]School of Life Sciences, University of Sussex, Brighton BN1 9QG, UK
[25]Department of Life Sciences, Imperial College London, Silwood Park, Ascot SL5 7PY, UK

*Correspondence: email: v.burton@nhm.ac.uk

Soil properties

Table S1: Soil properties obtained from the SoilGrids250m database [1] and their predicted effect on soil organism abundance.

Soil property	Expected effect on soil organism abundance
Organic carbon (tonnes / ha)	Positive: Soil organisms are generally less abundant in soils with low organic carbon [2, 3] or where organic carbon has been removed by intensive agriculture [4].
Bulk density (kg / m^3)	Negative: Soils with high bulk density are more compacted and so less hospitable [5, 6, 7].
pH in potassium chloride	Variable: relationship is expected to depend on taxa and land use; for example earthworms generally increase in abundance with pH [8, 9], while soil arthropod and nematode abundance correlate with pH in grassland in an opposite way [10]. Similarly, potworms (Enchytraeidae) dominate in low pH because of reduced competition from earthworms [11].
Available soil water capacity %<br>Saturated water %<br>Wilting point %	Positive: Most studies report soil organism abundance is positively correlated with soil moisture [3, 12, 13, 14], and sensitive to drought [15, 16] although responses may differ between spatial scales [17].
Clay %<br>Silt %<br>Sand %	Variable: Soils with higher clay content tend to have more organic material and better water retention, which are both linked to higher abundance of soil organisms. Soils with a high proportion of sand have lower water detention potential, so are less favourable [18]

Table S2: Numbers of sites within each biome for above-ground and soil biodiversity, and the weight given to above-ground sites within each biome.

Biome	Above-ground	Soil	Weight
Boreal Forests/Taiga	534	541	1.013
Temperate Conifer Forests	517	93	0.180
Temperate Broadleaf & Mixed Forests	7,160	4,579	0.640
Montane Grasslands & Shrublands	972	1	0.001
Temperate Grasslands, Savannas & Shrublands	1,138	22	0.019
Mediterranean Forests, Woodlands & Scrub	1,682	236	0.140
Deserts & Xeric Shrublands	236	37	0.157
Tropical & Subtropical Grasslands, Savannas & Shrublands	1,900	392	0.206
Tropical & Subtropical Coniferous Forests	300	67	0.223
Flooded Grasslands & Savannas	39	12	0.308
Tropical & Subtropical Dry Broadleaf Forests	368	144	0.391
Tropical & Subtropical Moist Broadleaf Forests	4,779	1,023	0.214
Mangroves	26	8	0.308

Table S3: Land use and land-use intensity classification definitions (after Hudson et al. 2014 [19])

Land use type	Minimal use	Light use	Intense use
Primary vegetation - native vegetation that is not known or inferred to have ever been completely destroyed, by human actions or by extreme natural events that do not normally play a role in ecosystem dynamics. Sites where primary vegetation has been destroyed by natural events that are part of the normal ecosystem dynamic (e.g. fire in Mediterranean ecosystems) remain as primary vegetation provided that regeneration is possible. Primary vegetation can be used by people, or been degraded, so long as it has never been completely destroyed.	Any disturbances identified are very minor (e.g. a trail or path) or very limited in the scope of their effect (e.g. hunting of a particular species of limited ecological importance).	One or more disturbances of moderate intensity (e.g. selective logging) or breadth of impact (e.g. bush meat extraction), which are not severe enough to markedly change the nature of the ecosystems. Primary sites in suburban settings are at least Light use.	One or more disturbances that are severe enough to markedly change the nature of the ecosystem; this includes clear felling of part of the site too recently for much recovery to have occurred. Primary sites in fully urban settings should be classed as Intense use.
Secondary vegetation - vegetation where the original primary vegetation was completely destroyed. This could be by human actions (including fire), and includes sites recovering to a natural state following a period of human-dominated land use. Secondary vegetation includes areas where humans have made an active attempt (through planting etc.) to restore an area to a more natural state. Although not managed as intensively as the human-dominated classes, such sites can be used by people in much the same way as primary vegetation sites.	As above	As above	As above

Plantation forest - previously cleared areas that people have planted with crop trees or crop shrubs for commercial or subsistence harvesting of wood and/or fruit.	Extensively managed or mixed timber, fruit/coffee, oil-palm or rubber plantations in which native understorey and/or other native tree species are tolerated, which are not treated with pesticide of fertiliser, and which have not been recently (<20 years) clear-felled.	Monoculture fruit/coffee/rubber plantations with limited pesticide input, or mixed species plantations with significant inputs. Monoculture timber plantations of mixed age with no recent (<20 years) clear-felling. Monoculture oil-palm plantations with no recent (<20 years) clear-felling.	Monoculture fruit/coffee/rubber plantations with significant pesticide input. Monoculture timber plantations with similarly aged trees or timber/oil-palm plantations with extensive recent (<20 years) clear-felling.
Cropland - land that people have planted with herbaceous crops, even if these crops will be fed to livestock once harvested.	Low-intensity farms, typically with small fields, mixed crops, crop rotation, little or no inorganic fertiliser use, little or no pesticide use, little or no ploughing, little or no irrigation, little or no mechanisation.	Medium intensity farming, typically showing some but not many of the following: large fields, annual ploughing, inorganic fertiliser application, pesticide application, irrigation, no crop rotation, mechanisation, monoculture crop. Organic farms in developed countries often fall within this category, as may high-intensity farming in developing countries.	High-intensity monoculture farming, typically showing many of the following features: large fields, annual ploughing, inorganic fertiliser application, pesticide application, irrigation, mechanisation, no crop rotation.

Pasture - land where livestock is known to be grazed regularly or permanently directly on the land.	Pasture with minimal input of fertiliser and pesticide, and will low stock density (not high enough to cause significant disturbance or stop regeneration of vegetation).	Pasture either with significant input of fertiliser or pesticide, or with high stock density (high enough to cause significant disturbance or to stop regeneration of vegetation).	Pasture with significant input of fertiliser or pesticide, and with high stock density (high enough to cause significant disturbance or to stop regeneration of vegetation).
Urban - areas with human habitation and/or buildings, where the primary vegetation has been removed, and where such vegetation is predominantly managed for civic or personal amenity.	Extensive managed green spaces, villages.	Suburban (e.g. gardens), or small managed or unmanaged green spaces in cities.	Fully urban with no significant green spaces.

Table S4: Data sources used in this analysis in addition to those in Hudson et al. (2016) [20] and which will be made available in a later addition to the PREDICTS database. For sources marked † data are available from the referenced paper. Figures here represent available data as curated by the PREDICTS team and will not necessarily match figures in the original papers.

Reference	Country	Biome	Studies	Sites	Samples	Taxa	Dates sampled
Albrecht and Haider (2013) [21]	Germany	Temperate Broadleaf & Mixed Forests	1	120	24,240	202	2008 - 2008
Blasi et al. (2013) [22]	Italy	Mediterranean Forests, Woodlands & Scrub	1	55	1,430	26	2007 - 2008
Bösing et al. (2014) [23]	South Africa	Deserts & Xeric Shrublands	1	22	242	11	2007 - 2007
Bogyó et al. (2015) [24]	Hungary	Temperate Broadleaf & Mixed Forests	1	12	168	14	2001 - 2001
Bone and Jones (2010) (unpublished) [25]	United Kingdom	Temperate Broadleaf & Mixed Forests	1	78	1,326	17	2010 - 2010
Bravo-Monroy et al. (2015) [26]	Colombia	Tropical & Subtropical Moist Broadleaf Forests	1	12	48	4	2011 - 2011
Burton (2015) (unpublished) [27]	United Kingdom	Temperate Broadleaf & Mixed Forests	1	33	363	11	2015 - 2015
Burton and Eggleton (2016) [28]	United Kingdom	Temperate Broadleaf & Mixed Forests	3	179	9,151	152	2013 - 2013
Calcaterra et al. (2010) [29]	Argentina	Flooded Grasslands & Savannas	1	45	2,250	50	2007 - 2008
Carpenter et al. (2012) (unpublished) [30]	United Kingdom	Temperate Broadleaf & Mixed Forests	1	105	6,195	59	2012 - 2012
Connelly et al. (2015) [31]	United States	Temperate Broadleaf & Mixed Forests	1	13	871	67	2012 - 2013
Cusser et al. (2015) [32]	United States	Temperate Grasslands, Savannas & Shrublands	1	9	450	50	2013 - 2013
Davies et al. (2015) [33]	Australia	Deserts & Xeric Shrublands	1	10	290	29	2013 - 2013
De Smedt et al. (2016) [34]	Belgium	Temperate Broadleaf & Mixed Forests	1	6	102	17	2011 - 2011
Eggleton et al. (2005) [35]	United Kingdom	Temperate Broadleaf & Mixed Forests	5	106	2,806	341	2001 - 2002
Farahat and Linderholm (2012) [36]	Egypt	Deserts & Xeric Shrublands	1	8	504	63	2010 - 2011
Farrell et al. (2011) [37]	United Kingdom	Temperate Broadleaf & Mixed Forests	1	20	760	38	2008 - 2009
Ferreira et al. (2013) [38]	Brazil	Tropical & Subtropical Moist Broadleaf Forests	1	7	98	14	2007 - 2008
Forrest et al. (2015) [39]	United States	Mediterranean Forests, Woodlands & Scrub	1	16	2,240	140	2002 - 2002
Gardner et al. (2007) [40]	Brazil	Tropical & Subtropical Moist Broadleaf Forests	1	3	159	53	2004 - 2005
Giangarelli et al. (2009) [41]	Brazil	Tropical & Subtropical Moist Broadleaf Forest	1	9	9	1	2001 - 2006
Hendrix et al. (2010) † [42]	United States	Temperate Broadleaf & Mixed Forests	1	5	365	73	2005 - 2005
Hodson et al. (2020) [43]	United Kingdom	Temperate Broadleaf & Mixed Forests	2	1,058	15,341	29	2013 - 2013
Holway and Suarez (2006) [44]	United States	Mediterranean Forests, Woodlands & Scrub	2	58	2,138	65	1998 - 1998
Horváth et al. (2012) [45]	Hungary	Temperate Broadleaf & Mixed Forests	1	6	414	69	2009 - 2009

Jackson et al. (2014) [46]	United States	Temperate Broadleaf & Mixed Forests	1	72	2,232	31	2010 - 2011
Jalilova et al. (2013) [47]	Kyrgyzstan	Temperate Grasslands, Savannas & Shrublands	1	5	115	23	2005 - 2005
Jha and Vandermeer (2010) [48]	Mexico	Tropical & Subtropical Moist Broadleaf Forest	1	7	315	45	2006 - 2006
Jones and Eggleton (2014) [9]	United Kingdom	Temperate Broadleaf & Mixed Forests	2	56	1,008	36	2009 - 2009
Kambach et al. (2012) [49]	Bolivia	Tropical & Subtropical Moist Broadleaf Forest	2	48	4,584	191	2011 - 2011
Kazerani et al. (2013) [50]	Iran	Temperate Conifer Forests	1	4	68	17	2013 - 2013
Knoll and Penatti (2012) [51]	Brazil	Tropical & Subtropical Grasslands, Savannas & Shrublands	1	5	49	10	1993 - 1999
Kosewska et al. (2013) [52]	Poland	Temperate Broadleaf & Mixed Forests	1	3	210	70	2009 - 2009
Kremen and M'Gonigle (2015) † [53]	United States	Mediterranean Forests, Woodlands & Scrub	1	15	1,455	97	2013 - 2013
Kutt and Fisher (2011) [54]	Australia	Tropical & Subtropical Grasslands, Savannas & Shrublands	1	38	6,080	160	2003 - 2003
Lacasella et al. (2015) [55]	Italy	Temperate Broadleaf & Mixed Forests	1	108	13,392	124	2009 - 2009
Lange et al. (2011) [56]	Germany	Temperate Broadleaf & Mixed Forests	1	48	1,728	36	2008 - 2008
Leong et al. (2014) [57]	United States	Mediterranean Forests, Woodlands & Scrub	1	12	180	15	2011 - 2011
Leong et al. (2016) [58]	United States	Mediterranean Forests, Woodlands & Scrub	1	35	3,184	91	2012 - 2012
Magura et al. (2008) [59]	Hungary	Temperate Broadleaf & Mixed Forests	1	12	708	59	2001 - 2002
Marinaro and Grau (2015) [60]	Argentina	Tropical & Subtropical Dry Broadleaf Forests	2	56	2,120	72	2009 - 2011
Massawe et al. (2012) [61]	United Republic of Tanzania	Tropical & Subtropical Grasslands, Savannas & Shrublands	1	10	110	11	2009 - 2009
Minor and Cianciolo (2007) [62]	United States	Temperate Broadleaf & Mixed Forests	1	14	1,512	108	1999 - 1999
Mulder et al. (2011) [63]	Netherlands	Temperate Broadleaf & Mixed Forests	4	2,040	396,270	777	2010 - 2010
Mumme et al. (2015) [64]	Indonesia	Tropical & Subtropical Moist Broadleaf Forest	1	32	28,672	896	2012 - 2012
Murvanidze et al. (2008) [65]	Georgia	Deserts & Xeric Shrublands	1	14	1,750	125	2008 - 2008
National Earthworm Recording Scheme (2017) [66]	United Kingdom	Temperate Broadleaf & Mixed Forests	1	117	2,691	23	2015 - 2015
Open Air Laboratories (OPAL) (2009) [67]	United Kingdom	Temperate Broadleaf & Mixed Forests	1	251	5,522	22	2009 - 2009
Pall et al. (2014) [68]	Argentina	Temperate Grasslands, Savannas & Shrublands	3	18	198	33	2008 - 2008
Ponge et al. (2015) [69]	France	Temperate Broadleaf & Mixed Forests	1	25	1,725	69	2006 - 2006
Shadhabuddin (2013) † [70]	Indonesia	Tropical & Subtropical Moist Broadleaf Forest	2	8	184	46	2012 - 2012
Soga et al. (2014) [71]	Japan	Temperate Broadleaf & Mixed Forests	2	42	1,596	76	2012 - 2012
Taboada et al. (2006) [72]	Spain	Mediterranean Forests, Woodlands & Scrub	1	20	1,220	61	2004 - 2004
Tonhasca et al. (2002) † [73]	Brazil	Tropical & Subtropical Moist Broadleaf Forest	1	9	189	21	1997 - 1999
Topp-Jørgensen et al. (2009) † [74]	United Republic of Tanzania	Tropical & Subtropical Moist Broadleaf Forest	2	6	27	9	2000 - 2000

Vu and Quang Vu (2011) [75]	Viet Nam	Tropical & Subtropical Moist Broadleaf Forest	1	4	448	112	2008 - 2009
Yan and Bao (2008) [76]	China	Montane Grasslands & Shrublands	1	26	6,032	232	2007 - 2007
Yan and Bao (2008a) [77]	China	Montane Grasslands & Shrublands	1	4	200	50	2006 - 2006
Yan and Bao (2011) [78]	China	Temperate Conifer Forests	1	4	236	59	2006 - 2006

Model structures

Structure of models fitted, response variable: log(total abundance+1), random effects structure: (1 — SS) + (1 — SSB). LUI = land use and intensity, BD = bulk density, OC = organic carbon.

Full model: LUI + CLAY + habitat layer + pH + OC + BD + LUI:habitat layer + LandUse:phkcl + LandUse:OC + LandUse:clay + LandUse:BD + habitat layer:phkcl + habitat layer:OC + habitat layer:clay + habitat layer:BD + LandUse:habitat layer:pH + LandUse:habitat layer:OC + LandUse:habitat layer:clay + LandUse:habitat layer:BD

Model for testing hypothesis 1) Soil and above-ground abundance respond differently to land-use: LUI + clay + habitat layer + pH + OC + BD + LandUse:pH + LandUse:OC + LandUse:CLAY + LandUse:BD + habitat layer:pH + habitat layer:OC + habitat layer:clay + habitat layer:BD

Model for testing hypothesis 2) soil physical properties mediate these differential responses: LUI + clay + habitat layer + pH + OC + BD + LUI:habitat layer + habitat layer:pH + habitat layer:OC + habitat layer:clay + habitat layer:BD

Model for testing hypothesis 3) modulation of abundance by soil properties is greatest for soil biota: LUI + clay + habitat layer + pH + OC + BD + LUI:habitat layer + LandUse:pH + LandUse:OC + LandUse:clay + LandUse:BD

Results

Figure S1: The explanatory power of models increases markedly with their complexity, from a model containing only habitat layer, through models considering only main effects of habitat layer and either land use intensity (LUI) or soil properties, through to additive and finally interactive models.

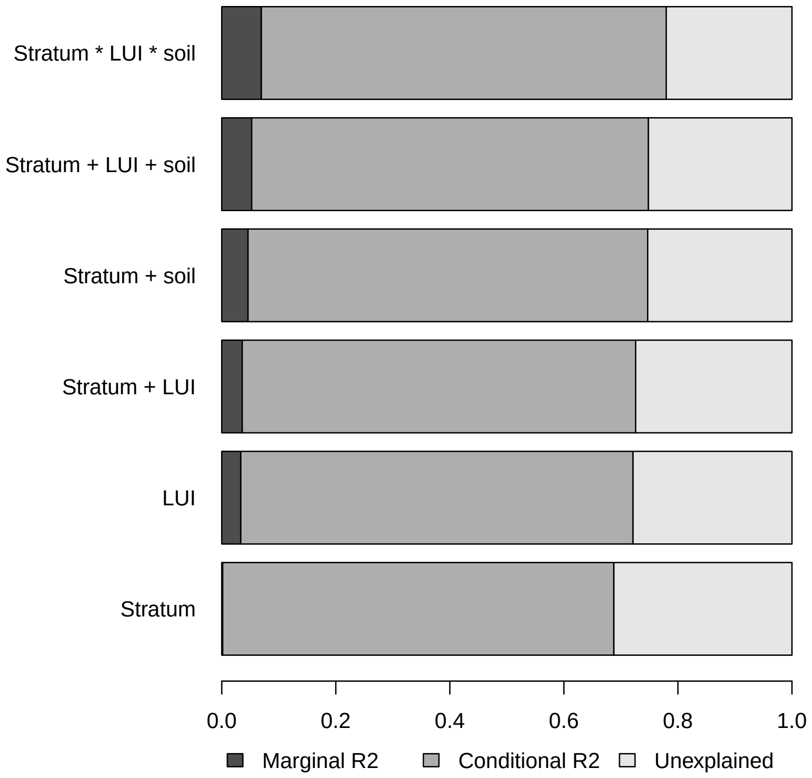

Unweighted model results

Figure S2: Response of above-ground (circles) and soil (triangles) organismal abundance to land-use type and intensity (from left to right within each land use: minimal, light and intense use) compared to abundance in primary vegetation in unweighted model. Responses have been back-transformed. Median values are used for other fixed effects. Error bars show 95% confidence intervals.

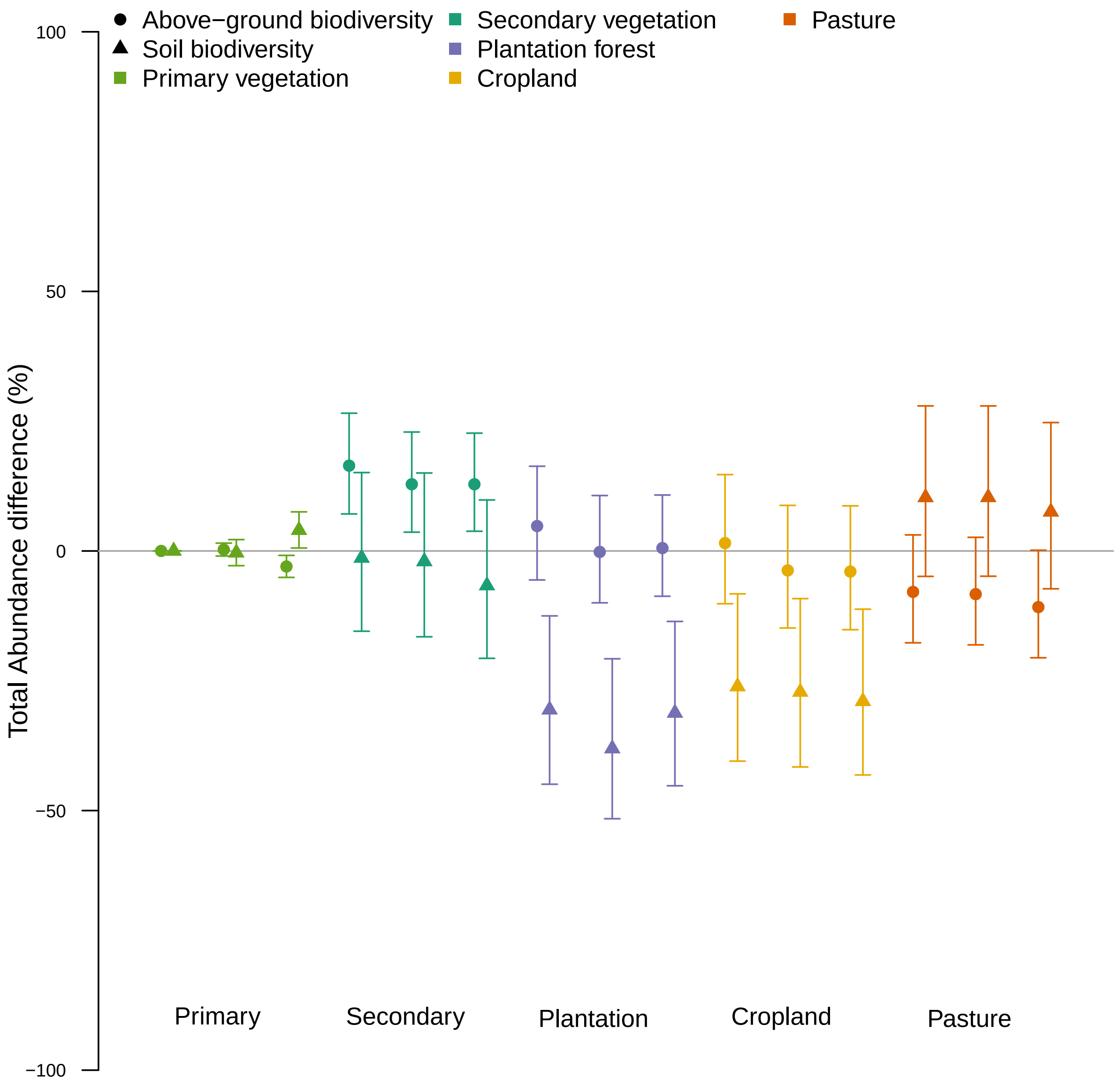

Figure S3: The (back-transformed) response of abundance to soil properties for five land uses for soil biodiversity (left) and above-ground biodiversity (right), with median values for other fixed effects. Shading spans ±0.5 standard errors, and rugs along the x axes show the values of the explanatory variables represented in the data set used for modelling.

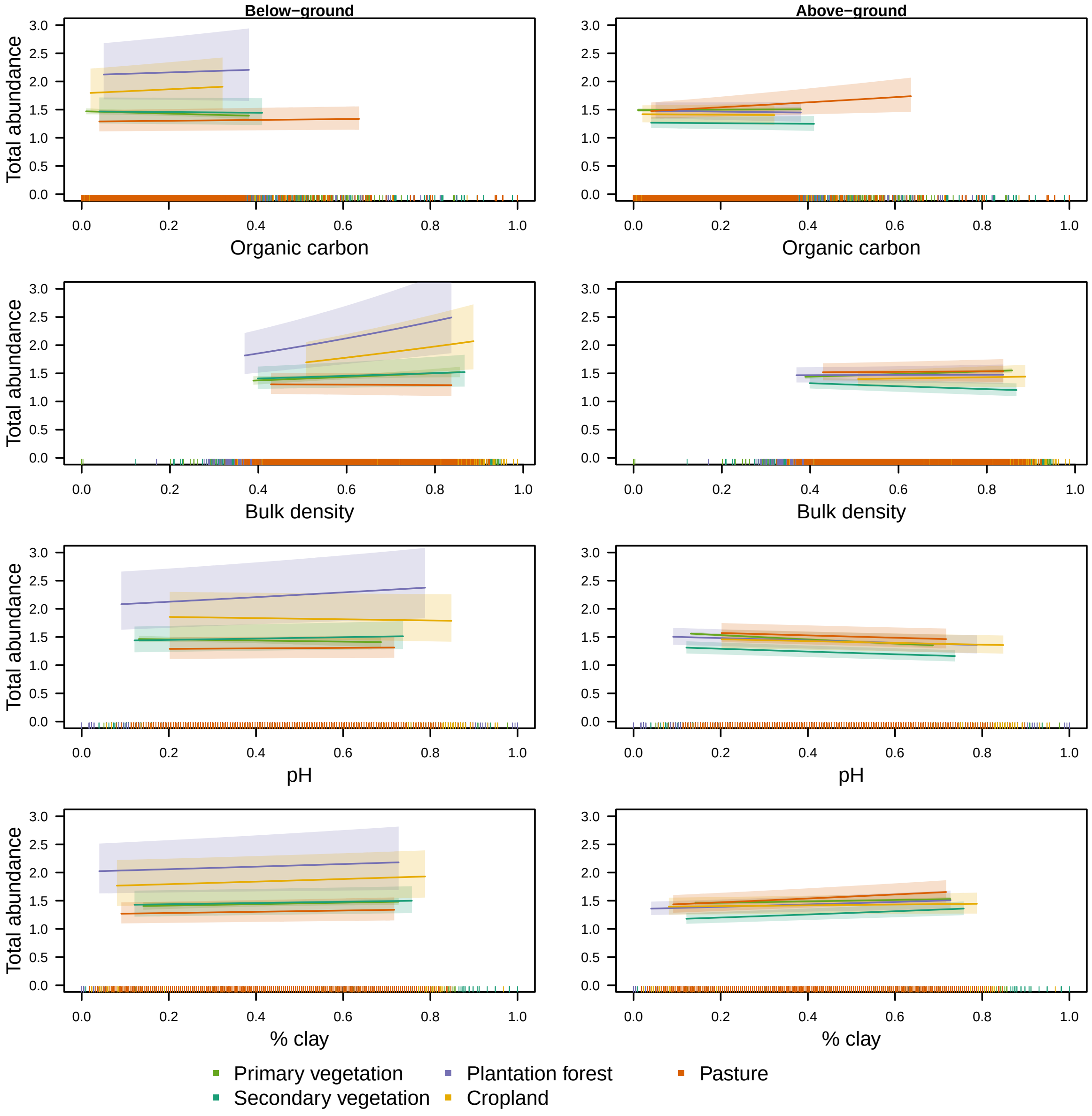

Figure S4: The explanatory power of the unweighted models increases markedly with their complexity, from a model containing only habitat layer, through models considering only main effects of habitat layer and either land use intensity (LUI) or soil properties, through to additive and finally interactive models.

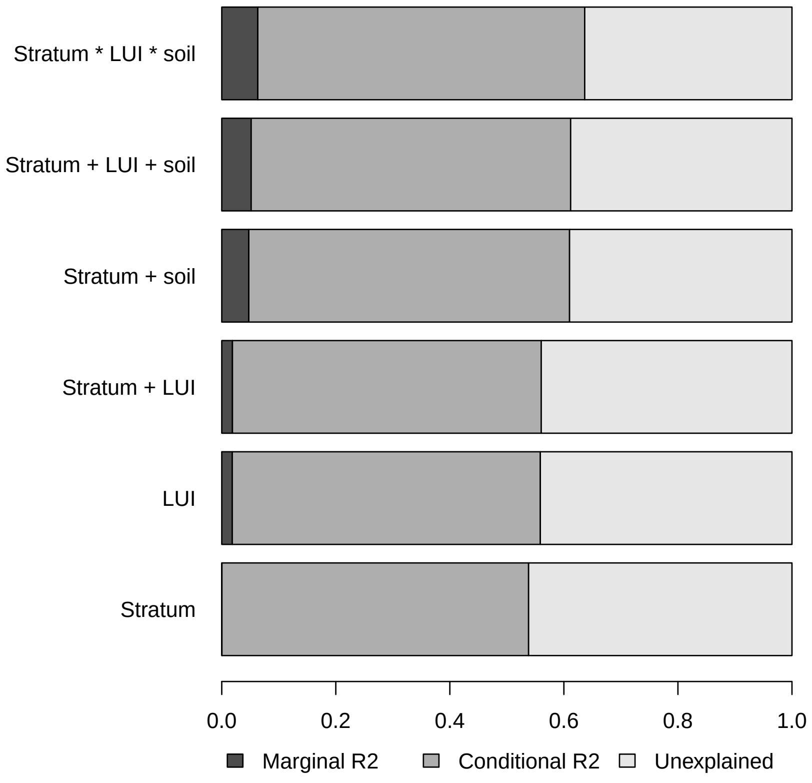

Invertebrate-only model results

Figure S5: Response of above-ground (circles) and soil (triangles) invertebrate abundance to land-use type and intensity (from left to right within each land use: minimal, light and intense use) compared to abundance in primary vegetation using invertebrate-only subset of the dataset. Responses have been back-transformed. Median values are used for other fixed effects. Error bars show 95% confidence intervals.

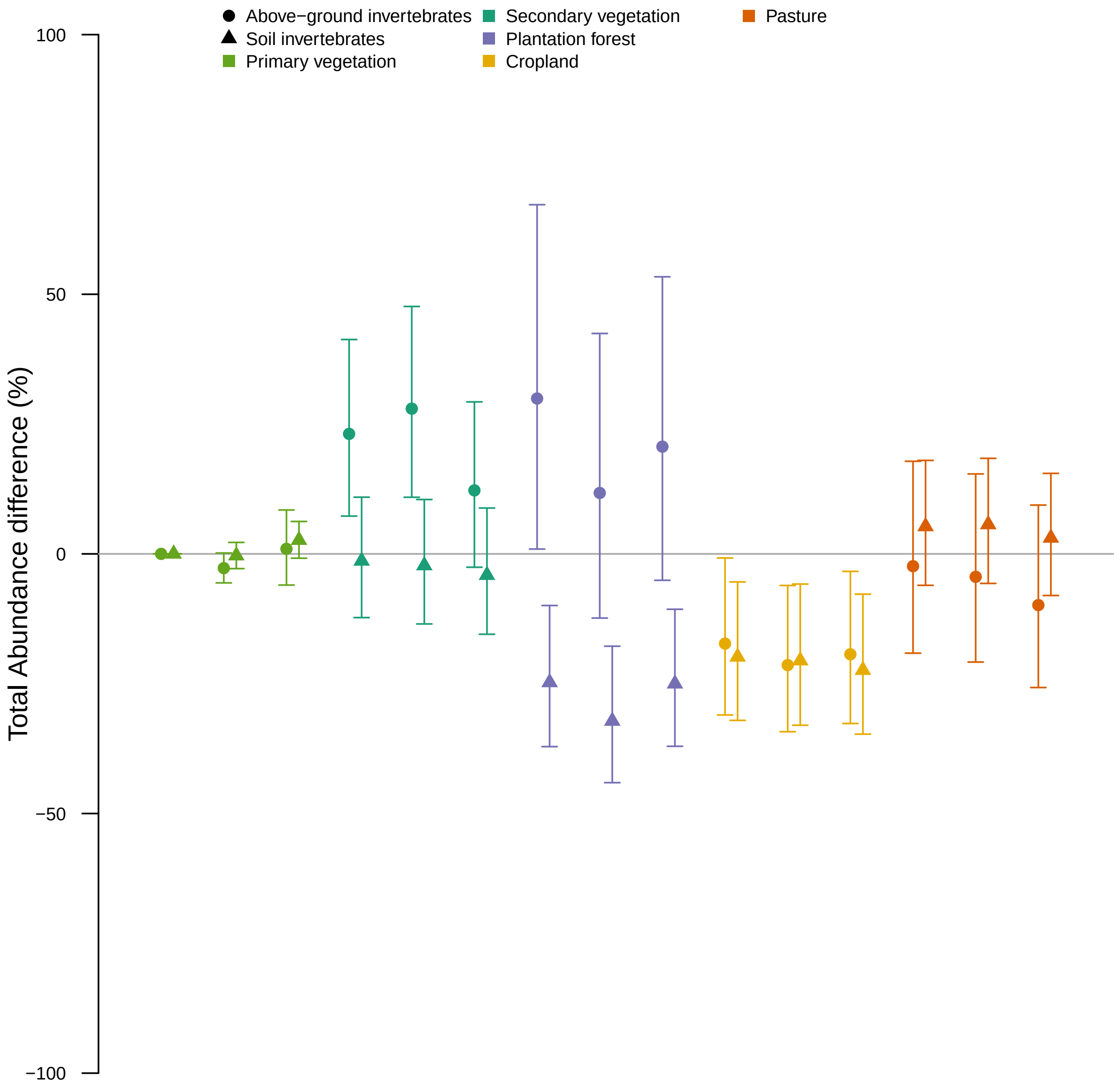

Figure S6: The (back-transformed) response of abundance to soil properties for five land uses for soil invertebrate biodiversity (left) and above-ground invertebrate biodiversity (right), with median values for other fixed effects. Shading spans ±0.5 standard errors, and rugs along the x axes show the values of the explanatory variables represented in the data set used for modelling.

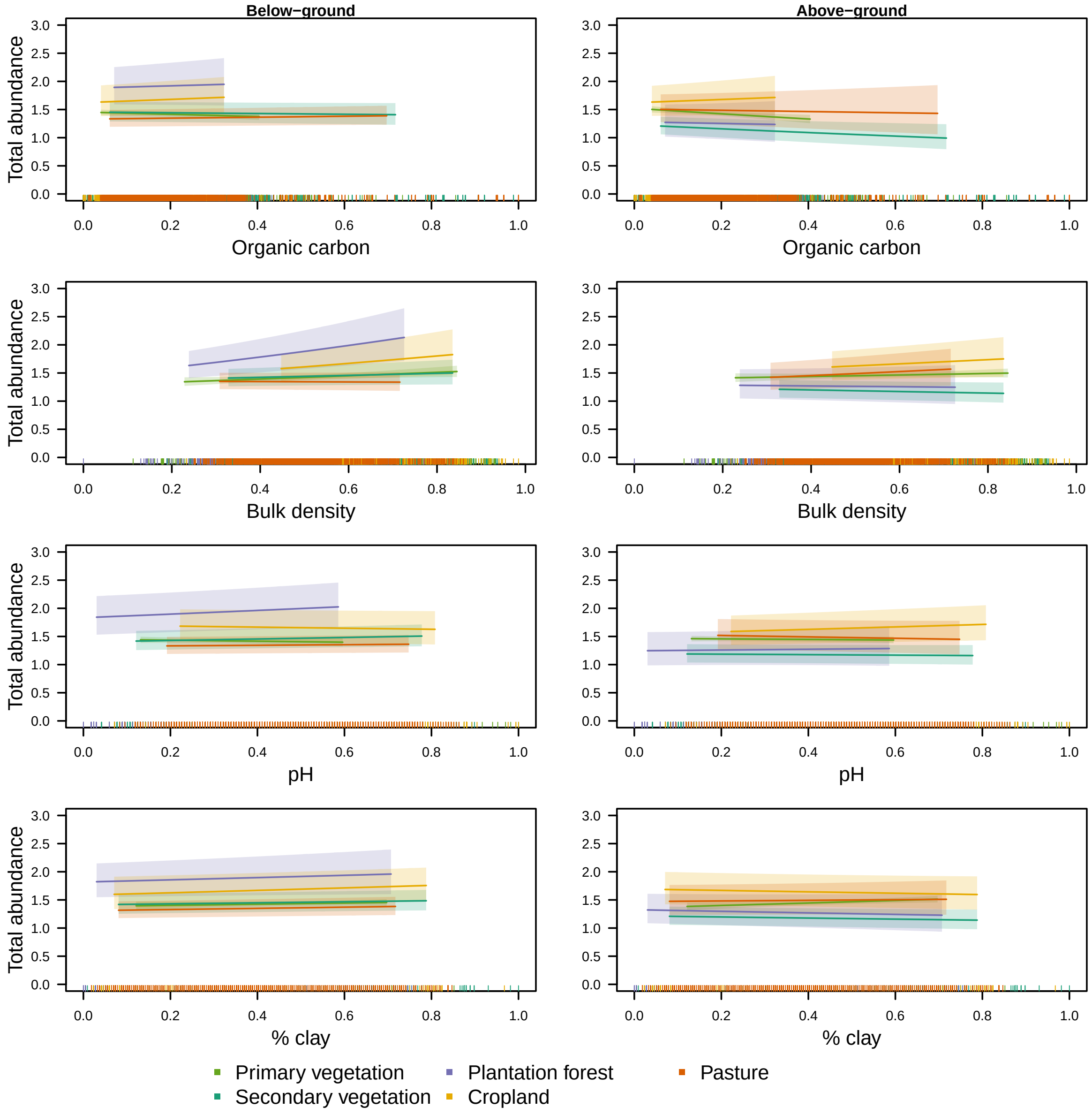

References

[1] Hengl T, Mendes De Jesus J, Heuvelink GBM, Gonzalez MR, Kilibarda M, Blagotí A, et al. SoilGrids250m: Global Gridded Soil Information Based on Machine Learning. PLOS One. 2017;12(2):1–40.

[2] Johnston ASA, Sibly RM. Multiple environmental controls explain global patterns in soil

animal communities. Oecologia. 2020;192(4):1047–1056. Available from: https://doi.org/10.1007/s00442-020-04640-w.

[3] Zhao K, Jing X, Sanders NJ, Chen L, Shi Y, Flynn DFB, et al. On the controls of abundance for soil-dwelling organisms on the Tibetan Plateau. Ecosphere. 2017;8(7):1–14.

[4] Blakemore R. Critical Decline of Earthworms from Organic Origins under Intensive, Humic SOM-Depleting Agriculture. Soil Systems. 2018;2(2):1–28.

[5] Beylich A, Oberholzer HR, Schrader S, Höper H, Wilke BM. Evaluation of soil compaction effects on soil biota and soil biological processes in soils. Soil and Tillage Research. 2010;109(2):133–143.

[6] Nielsen UN, Ayres E, Wall DH, Li G, Bardgett RD, Wu T, et al. Global-scale patterns of assemblage structure of soil nematodes in relation to climate and ecosystem properties. Global Ecology and Biogeography. 2014;23(9):968–978.

[7] Whalley WR, Dumitru E, Dexter AR. Biological effects of soil compaction. Soil and Tillage Research. 1995;35(1-2):53–68.

[8] Edwards CA, Bohlen PJ. Biology and Ecology of Earthworms. 3rd ed. Chapman and Hall; 1996.

[9] Jones DT, Eggleton P. Earthworms in England: distribution, abundance and habitats (Natural England Commissioned Report NECR145). Natural England; 2014. Available from: http://publications.naturalengland.org.uk/file/5824256822738944.

[10] Mulder C, Elser JJ. Soil acidity, ecological stoichiometry and allometric scaling in grassland food webs. Global Change Biology. 2009;15(11):2730–2738.

[11] Raty M, Huhta V. Earthworms and pH affect communities of nematodes and enchytraeids in forest soil. Biology and Fertility of Soils. 2003;38(1):52–58.

[12] Eggleton P, Inward K, Smith J, Jones DT, Sherlock E. A six year study of earthworm (Lumbricidae) populations in pasture woodland in southern England shows their responses to soil temperature and soil moisture. Soil Biology and Biochemistry. 2009;41(9):1857–1865.

[13] Phillips HRP, Beaumelle L, Tyndall K, Burton VJ, Cameron EK, Eisenhauer N, et al. The effects of global change on soil faunal communities: a meta-analytic approach. Research

Ideas and Outcomes. 2019;5(e36427). Available from: https://riojournal.com/article/36427/.

[14] van den Hoogen J, Geisen S, Routh D, Ferris H, Traunspurger W, Wardle DA, et al. Soil nematode abundance and functional group composition at a global scale. Nature. 2019;572(7768):194–198.

[15] Peng Y, Peñuelas J, Vesterdal L, Yue K, Peguero G, Fornara DA, et al. Responses of soil fauna communities to the individual and combined effects of multiple global change factors. Ecology Letters. 2022 7;(May):1–13. Available from: https://onlinelibrary.wiley.com/doi/10.1111/ele.14068.

[16] Blankinship JC, Niklaus PA, Hungate BA. A meta-analysis of responses of soil biota to global change. Oecologia. 2011;165(3):553–565. Available from: http://link.springer.com/10.1007/s00442-011-1909-0.

[17] Sylvain ZA, Wall DH, Cherwin KL, Peters DPC, Reichmann LG, Sala OE. Soil animal responses to moisture availability are largely scale, not ecosystem dependent: insight from a cross-site study. Global Change Biology. 2014;20(8):2631–2643.

[18] Orgiazzi A, Bardgett RD, Barrios E, Behan-Pelletier V, Briones MJI, Chotte JL, et al., editors. Global Soil Biodiversity Atlas. Luxembourg: European Commission, Publications Office of the European Union; 2016.

[19] Hudson LN, Newbold T, Contu S, Hill SLL, Lysenko I, De Palma A, et al. The PREDICTS database: a global database of how local terrestrial biodiversity responds to human impacts. Ecology and Evolution. 2014;4(24):4701–4735.

[20] Hudson LN, Newbold T, Contu S, Hill SLLL, Lysenko I, De Palma A, et al. The database of the PREDICTS (Projecting Responses of Ecological Diversity In Changing Terrestrial Systems) project. Ecology and Evolution. 2016;7(1):145–188.

[21] Albrecht H, Haider S. Species diversity and life history traits in calcareous grasslands vary along an urbanization gradient. Biodiversity and Conservation. 2013;22(10):2243–2267.

[22] Blasi S, Menta C, Balducci L, Conti FD, Petrini E, Piovesan G. Soil microarthropod communities from Mediterranean forest ecosystems in Central Italy under different disturbances. Environmental Monitoring and Assessment. 2013;185(2):1637–1655.

[23] Bösing BM, Haarmeyer DH, Dengler J, Ganzhorn JU, Schmiedel U. Effects of livestock grazing and habitat characteristics on small mammal communities in the Knersvlakte, South Africa. Journal of Arid Environments. 2014;104(104):124–131.

[24] Bogyó D, Magura T, Simon E, Tóthmérész B. Millipede (Diplopoda) assemblages alter drastically by urbanisation. Landscape and Urban Planning. 2015;133:118–126.

[25] Bone J, Jones DT. Shropshire earthworm hotspots project (unpublished data); 2010.

[26] Bravo-Monroy L, Tzanopoulos J, Potts SG. Ecological and social drivers of coffee pollination in Santander, Colombia. Agriculture, Ecosystems & Environment. 2015;211:145–154.

[27] Burton VJ. Purbeck heathland restoration soil biodiversity sampling (unpublished data); 2015.

[28] Burton VJ, Eggleton P. Microhabitat heterogeneity enhances soil macrofauna and plant species diversity in an Ash – Field Maple woodland. European Journal of Soil Biology. 2016;75:97–106.

[29] Calcaterra LA, Cabrera SM, Cuezzo F, Peréz IJ, Briano JA. Habitat and Grazing Influence on Terrestrial Ants in Subtropical Grasslands and Savannas of Argentina. Annals of the Entomological Society of America. 2010;103(4):635–646.

[30] Carpenter D, Holland J, Eggleton P, Smith B. A comparison of the effect of managed burning and vegetation cutting on biodiversity in the New Forest (unpublished data); 2012.

[31] Connelly H, Poveda K, Loeb G. Landscape simplification decreases wild bee pollination services to strawberry. Agriculture, Ecosystems and Environment. 2015;211(December):51–56. Available from: http://dx.doi.org/10.1016/j.agee.2015.05.004.

[32] Cusser S, Neff JL, Jha S. Land use change and pollinator extinction debt in exurban landscapes. Insect Conservation and Diversity. 2015;8(6):562–572.

[33] Davies SJJF, Kenny SA, Walsh TFM. The population dynamics of some arid zone plants during 46 years of grazing on Mileura Station, Western Australia. The Rangeland Journal. 2015;37(4):367.

[34] De Smedt P, Wuyts K, Baeten L, De Schrijver A, Proesmans W, De Frenne P, et al. Complementary distribution patterns of arthropod detritivores (woodlice and millipedes)

along forest edge-to-interior gradients. Insect Conservation and Diversity. 2016;9(5):456–469.

[35] Eggleton P, Vanbergen AJ, Jones DT, Lambert MC, Rockett C, Hammond PM, et al. Assemblages of soil macrofauna across a Scottish land-use intensification gradient: influences of habitat quality, heterogeneity and area. Journal of Applied Ecology. 2005;42(6):1153–1164.

[36] Farahat E, Linderholm HW. Ecological impacts of desert plantation forests on biodiversity. African Journal of Ecology. 2012;50(3):308–318.

[37] Farrell M, Hill PW, Farrar J, Bardgett RD, Jones DL. Seasonal variation in soluble soil carbon and nitrogen across a grassland productivity gradient. Soil Biology and Biochemistry. 2011;43(4):835–844.

[38] Ferreira RP, Martins C, Dutra MC, Mentone CB, Antonini Y. Old Fragments of Forest Inside an Urban Area Are Able to Keep Orchid Bee (Hymenoptera: Apidae: Euglossini) Assemblages? The Case of a Brazilian Historical City. Neotropical Entomology. 2013;42(5):466–473.

[39] Forrest JRK, Thorp RW, Kremen C, Williams NM. Contrasting patterns in species and functional-trait diversity of bees in an agricultural landscape. Journal of Applied Ecology. 2015;52(3):706–715.

[40] Gardner TA, Ribeiro-Júnior MA, Barlow J, Ávila-Pires TCS, Hoogmoed MS, Peres CA. The Value of Primary, Secondary, and Plantation Forests for a Neotropical Herpetofauna. Conservation Biology. 2007;21(3):775–787.

[41] Giangarelli DC, Freiria GA, Colatreli OP, Suzuki KM, Sofia SH. Eufriesea violacea (Blanchard) (Hymenoptera: Apidae): an orchid Bee apparently sensitive to size reduction in forest patches. Neotropical Entomology. 2009;38(5):610–615.

[42] Hendrix SD, Kwaiser KS, Heard SB. Bee communities (Hymenoptera: Apoidea) of small Iowa hill prairies are as diverse and rich as those of large prairie preserves. Biodiversity and Conservation. 2010;19(6):1699–1709.

[43] Hodson ME, Corstanje R, Jones DT, Witton J, Burton VJ, Sloan T, et al. Earthworm distributions are not driven by measurable soil properties. Do they really indicate soil quality? PLOS ONE. 2021 8;16(8):e0241945.

[44] Holway DA, Suarez AV. Homogenization of ant communities in mediterranean California: The effects of urbanization and invasion. Biological Conservation. 2006;127(3):319–326.

[45] Horváth R, Magura T, Tóthmérész B. Ignoring ecological demands masks the real effect of urbanization: a case study of ground-dwelling spiders along a rural–urban gradient in a lowland forest in Hungary. Ecological Research. 2012 11;27(6):1069–1077. Available from: http://doi.wiley.com/10.1007/s11284-012-0988-7.

[46] Jackson MM, Turner MG, Pearson SM. Logging Legacies Affect Insect Pollinator Communities in Southern Appalachian Forests. Southeastern Naturalist. 2014;13(2):317–336.

[47] Jalilova G, Groot Jd, Vacik H. Evaluating the effects of habitats on birds in the walnut fruit forests: a case study from Kyrgyzstan. Biodiversity. 2013;14(2):97–110.

[48] Jha S, Vandermeer JH. Impacts of coffee agroforestry management on tropical bee communities. Biological Conservation. 2010;143(6):1423–1431.

[49] Kambach S, Guerra F, Beck S, Hensen I, Schleuning M. Human-Induced Disturbance Alters Pollinator Communities in Tropical Mountain Forests. Diversity. 2012;5(4):1–14.

[50] Kazerani F, Khaghaninia S, Grichanov I. Diversity of the genus Dolichopus Latreille in three different habitats of East Azerbaijan Province, with new records for Iran. Arxius de Miscel·lània Zoològica. 2013;11:134–152.

[51] Knoll FdRN, Penatti NC. Habitat Fragmentation Effects on the Orchid Bee Communities in Remnant Forests of Southeastern Brazil. Neotropical Entomology. 2012;41(5):355–365.

[52] Kosewska A, Nietupski M, Damszel M. Role of urban forests as a source of diversity of carabids (Coleoptera: Carabidae) in urbanised areas. Baltic Journal of Coleopterology. 2013;13(1):27–40.

[53] Kremen C, M'Gonigle LK. Small-scale restoration in intensive agricultural landscapes supports more specialized and less mobile pollinator species. Journal of Applied Ecology. 2015;52(3):602–610.

[54] Kutt AS, Fisher A. Increased grazing and dominance of an exotic pasture (Bothriochloa pertusa) affects vertebrate fauna species composition, abundance and habitat in savanna woodland. The Rangeland Journal. 2011;33(1):49.

[55] Lacasella F, Gratton C, De Felici S, Isaia M, Zapparoli M, Marta S, et al. Asymmetrical responses of forest and “beyond edge” arthropod communities across a forest–grassland ecotone. Biodiversity and Conservation. 2015;24(3):447–465.

[56] Lange M, Weisser WW, Gossner MM, Kowalski E, Türke M, Joner F, et al. The impact of forest management on litter-dwelling invertebrates: a subtropical–temperate contrast. Biodiversity and Conservation. 2011;20(10):2133–2147.

[57] Leong M, Kremen C, Roderick GK. Pollinator Interactions with Yellow Starthistle (Centaurea solstitialis) across Urban, Agricultural, and Natural Landscapes. Plos One. 2014;9(1):10.

[58] Leong M, Ponisio LC, Kremen C, Thorp RW, Roderick GK. Temporal dynamics influenced by global change: bee community phenology in urban, agricultural, and natural landscapes. Global Change Biology. 2016;22(3):1046–1053.

[59] Magura T, Lövei GL, Tóthmérész B. Time-consistent rearrangement of carabid beetle assemblages by an urbanisation gradient in Hungary. Acta Oecologica. 2008;34(2):233–243.

[60] Marinaro S, Grau RH. Comparison of animal biodiversity in three livestock systems of open environments of the semi-arid Chaco of Argentina. Rangeland Journal. 2015;37(5):497–505.

[61] Massawe AW, Makundi RH, Mulungu LS, Katakweba A, Shayo TN. Breeding Dynamics of Rodent Species Inhabiting Farm—Fallow Mosaic Fields in Central Tanzania. African Zoology. 2012;47(1):128–137.

[62] Minor MAA, Cianciolo JMM. Diversity of soil mites (Acari: Oribatida, Mesostigmata) along a gradient of land use types in New York. Applied Soil Ecology. 2007 1;35(1):140–153. Available from: https://www.sciencedirect.com/science/article/pii/S0929139306001193.

[63] Mulder C, Boit A, Bonkowski M, De Ruiter PC, Mancinelli G, Van der Heijden MGAA, et al. A Belowground Perspective on Dutch Agroecosystems: How Soil Organisms Interact

to Support Ecosystem Services. In: Advances in Ecological Research. vol. 44. 1st ed. Elsevier Academic Press; 2011. p. 277–357.

[64] Mumme S, Jochum M, Brose U, Haneda NF, Barnes AD. Functional diversity and stability of litter-invertebrate communities following land-use change in Sumatra, Indonesia. Biological Conservation. 2015;191:750–758.

[65] Murvanidze, M Murvanidze, Arabuli T, Bagaturia N, Eliava I, Kvavadze E, et al. The nematodes and oribatid mites as indicators of urban environment. Proceedings of the institute of Zoology. 2008;XXIII(March 2016):180–193.

[66] National Earthworm Recording Scheme. National Earthworm Recording Scheme Records (Great Britain) occurrence dataset; 2017.

[67] Open Air Laboratories (OPAL). OPAL Soil and Earthworm Survey; 2009. Available from: https://www.opalexplorenature.org/dataexplorer/surveytype/soilsurvey.

[68] Pall J, Kihn RG, Bernados J, Quiran EM. Biodiversity of the Ants (Insecta: Hymenoptera) Associated With the Center Agroecosystems of Argentina. Munis Entomology & Zoology. 2014;9(1):170–177.

[69] Ponge JF, Salmon S, Benoist A, Geoffroy JJ. Soil Macrofaunal Communities are Heterogeneous in Heathlands with Different Grazing Intensity. Pedosphere. 2015;25(4):524–533.

[70] Shadhabuddin. Contribution of Alpha and Beta Diversity Across Land-Use Type to the Regional Diversity of Dung Beetles in Central Sulawesi. HAYATI Journal of Biosciences. 2013;20(2):72–79.

[71] Soga M, Yamaura Y, Koike S, Gaston KJ. Woodland remnants as an urban wildlife refuge: A cross-taxonomic assessment. Biodiversity and Conservation. 2014;23(3):649–659.

[72] Taboada A, Kotze DJ, Tárrega R, Salgado JM. Traditional forest management: Do carabid beetles respond to human-created vegetation structures in an oak mosaic landscape? Forest Ecology and Management. 2006;237(1-3):436–449.

[73] Tonhasca A, Blackmer JL, Albuquerque GS. Abundance and Diversity of Euglossine Bees in the Fragmented Landscape of the Brazilian Atlantic Forest1. Biotropica. 2002;34(3):416–422.

[74] Topp-Jørgensen E, Reinhardt Nielsen M, Marshall AR, Pedersen U. Relative densities of mammals in response to different levels of bushmeat hunting in the Udzungwa Mountains, Tanzania. densities of mammals in response to different levels of bushmeat hunting in the Udzungwa. Tropical Conservation Science. 2009;2(1):70–87. Available from: www.tropicalconservationscience.org.

[75] Vu LV, Quang Vu C. Diversity Pattern of Butterfly Communities (Lepidoptera, Papilionoidae) in Different Habitat Types in a Tropical Rain Forest of Southern Vietnam. ISRN Zoology. 2011;2011:1–8.

[76] Yan X, Bao W. Structure and species composition of ground bryophyte community of high-altitude young silvicultural cutovers in Rangtang County, China: evaluation on effects of clear-cutting and silvicultural management. Journal of Plant Ecology (Chinese Version). 2008;32(4):815–824.

[77] Yan X, Bao W. Evaluation of species composition and development of bryophyte community during early natural recovery progress of high-altitude spruce cutovers. Biodiversity Science. 2008;16(2):110.

[78] Yan X, Bao W. Ground bryophyte diversity in secondary birch forests in western Sichuan, China. Biodiversity Science. 2011;19(3):327–334.
